# Randomized Controlled Trials to Define Viral Load Thresholds for Cytomegalovirus Pre-Emptive Therapy

**DOI:** 10.1371/journal.pone.0163722

**Published:** 2016-09-29

**Authors:** Paul D. Griffiths, Emily Rothwell, Mohammed Raza, Stephanie Wilmore, Tomas Doyle, Mark Harber, James O’Beirne, Stephen Mackinnon, Gareth Jones, Douglas Thorburn, Frank Mattes, Gaia Nebbia, Sowsan Atabani, Colette Smith, Anna Stanton, Vincent C. Emery

**Affiliations:** 1 Centre for Virology University College London Medical School, London, United Kingdom; 2 Renal Transplant Unit Royal Free Hospital, London, United Kingdom; 3 The Royal Free Sheila Sherlock Liver Centre, Royal Free Hospital, London, United Kingdom; 4 Department of Haematology, University College London, London, United Kingdom; 5 Research Department of Infection and Population Health, University College London, London, United Kingdom; Universita degli Studi di Roma Tor Vergata, ITALY

## Abstract

**Background:**

To help decide when to start and when to stop pre-emptive therapy for cytomegalovirus infection, we conducted two open-label randomized controlled trials in renal, liver and bone marrow transplant recipients in a single centre where pre-emptive therapy is indicated if viraemia exceeds 3000 genomes/ml (2520 IU/ml) of whole blood.

**Methods:**

Patients with two consecutive viraemia episodes each below 3000 genomes/ml were randomized to continue monitoring or to immediate treatment (Part A). A separate group of patients with viral load greater than 3000 genomes/ml was randomized to stop pre-emptive therapy when two consecutive levels less than 200 genomes/ml (168 IU/ml) or less than 3000 genomes/ml were obtained (Part B). For both parts, the primary endpoint was the occurrence of a separate episode of viraemia requiring treatment because it was greater than 3000 genomes/ml.

**Results:**

In Part A, the primary endpoint was not significantly different between the two arms; 18/32 (56%) in the monitor arm had viraemia greater than 3000 genomes/ml compared to 10/27 (37%) in the immediate treatment arm (p = 0.193). However, the time to developing an episode of viraemia greater than 3000 genomes/ml was significantly delayed among those randomized to immediate treatment (p = 0.022). In Part B, the primary endpoint was not significantly different between the two arms; 19/55 (35%) in the less than 200 genomes/ml arm subsequently had viraemia greater than 3000 genomes/ml compared to 23/51 (45%) among those randomized to stop treatment in the less than 3000 genomes/ml arm (p = 0.322). However, the duration of antiviral treatment was significantly shorter (p = 0.0012) in those randomized to stop treatment when viraemia was less than 3000 genomes/ml.

**Discussion:**

The results illustrate that patients have continuing risks for CMV infection with limited time available for intervention. We see no need to alter current rules for stopping or starting pre-emptive therapy.

## Introduction

Opportunistic infection represents a significant problem after transplantation. Cytomegalovirus (CMV) is the most common opportunist and may produce serious end organ disease if not controlled by deploying antiviral drugs for prophylaxis or pre-emptive therapy.[1–3] In transplant recipients with CMV infection, CMV disease occurs in those with high CMV viral loads so regular monitoring of CMV DNA in blood (viraemia) coupled with treatment of low viral loads is effective at preventing disease.[4–6] Indeed, end organ disease is now too uncommon to be used as the primary end point for randomized clinical trials of antiviral drugs. Historically at our institution, patients were given pre-emptive therapy on the basis of two consecutive positive CMV PCR results as detected by a qualitative PCR technique with a lower limit of detection of 200 genomes/ml whole blood.[7] With the introduction of real time PCR in 2003, it became possible to incorporate quantitative data into the clinical management of individual patients while maintaining the same lower limit of detection.[8]

Our natural history data showed that patients with CMV end organ disease had CMV PCR viraemia ranging from 14,000 to 203 million (median 175,500) genomes/ml blood.[4,9] The lower 95% confidence interval (CI) of the median of viral loads was 37,000 genomes/ml blood and we aimed to initiate therapy in time to prevent CMV viral load reaching this value. To give a margin of safety, bearing in mind the 1 day average doubling-time of CMV[10] and the timing of sampling (twice-weekly), the protocols for patient management were modified to initiate therapy once the viral load in blood increased above 3,000 genomes/ml. Coincident with this policy change to real-time quantitative PCR monitoring, we established two open-label randomized controlled trials to determine if exposure to the drugs used (ganciclovir, valganciclovir or foscarnet) could be reduced further. Before 2003, all patients with two consecutive positive CMV viral loads (including those between 200 and 3,000 genomes/ml) received pre-emptive treatment because the previous PCR assay did not give a quantitative result. Under the new policy, only those with viral loads greater than 3000 genomes/ml would be treated. We therefore conducted a randomized controlled trial to determine whether those patients with 'low level' viral loads (between 200 and 3,000 genomes/ml) could be monitored as opposed to starting pre-emptive therapy immediately. In parallel, we initiated a separate randomized controlled trial to determine whether the patients given pre-emptive therapy because of high-level viral loads (above 3,000 genomes/ml blood) could stop pre-emptive therapy earlier, potentially maximizing the benefits of therapy while minimizing its risks. We chose to randomize patients to stop treatment when they had two consecutive blood samples with viral loads below the level of the assay cut-off (200 genomes/ml) or two blood results below a level of 3000 genomes/ml (the level at which treatment was initiated for all). We hypothesized that, as viral loads would be decreasing in patients because of the effect of the drugs used for pre-emptive therapy, together with a contribution from the immune system of the patients, they may not require extended treatment once viral loads reduced below a critical point.

These randomized controlled trials were designed from the perspective of a diagnostic virology laboratory aiming to provide advice to our clinical colleagues on when to start and stop pre-emptive therapy. The viral load parameters for the three transplant patient groups studied here (bone marrow transplant, renal transplant, liver transplant) are slightly different in that the bone marrow patients get CMV end-organ disease at a lower level than do the solid organ transplant recipients.[4] However, the values found soon after transplant by monitoring are sufficiently similar to allow patients to be combined for the purposes of randomized controlled trials, using stratification within each patient group and we have previously used this approach to conduct a randomized controlled trial of ganciclovir versus ganciclovir/foscarnet in patients from all three transplant groups.[7]

## Materials and Methods

### Patients

Patients undergoing CMV surveillance in a single institution because of allogeneic transplantation of kidney, liver or haematopoietic stem cells were studied.[8,11] Exclusions for both parts included simultaneous transplantation of more than one organ or previous CMV viraemia or disease. An exclusion for Part A was patients requiring pre-emptive therapy because their viral load was greater than 3,000 genomes/ml. The eligibility criteria did not change during the study.

### Polymerase chain reaction and surveillance

Our strategy for CMV surveillance is described elsewhere.[8] Briefly, samples of whole blood were collected twice a week from in-patients and out-patients for the first 60 days post transplant, then once a week with the objective of monitoring CMV replication for the first 90 days after transplantation. Further samples were collected from CMV viraemic patients to follow replication episodes through to resolution. Whole blood was used rather than plasma for these studies because the natural history data used to define cut-off values was derived from analyses of whole blood. [4, 7–10] Because the CMV DNA results were used for patient management, samples sent by clinicians were tested and reported with some expected long durations of viraemia e.g. median 64 days (range 1–323) for D+R- renal transplants. [8]

The real time PCR method used a Taqman probe and the ABI7700 thermal cycler as described elsewhere.[12] The values of 200 and 3000 genomes/ml given here convert to 168 and 2520 international units/ml respectively using the WHO international standard.[13]

Doubling times of CMV viraemia and decline rates following therapy were calculated using a standard exponential function.[10] The nadir viral load for each patient having a second episode was computed from the intersection of the plot of decline versus plot of recurrence of viral load extrapolated from the linear regression fitting of the log10 viral load time plots (decline and recurrence).

### Design of the two randomized controlled trials

Randomization employed random number tables administered by means of sequentially numbered sealed envelopes prepared by the first author, one set for each patient group, to allow stratification by patient type. The randomization did not use pre-specified block sizes and the allocation ratio was 1:1. The study was implemented by medical staff in the Centre for Virology. As soon as a PCR result indicated that a patient was potentially eligible, the result was discussed with their attending physician. If the patient was considered well enough to be approached, the study objectives were explained and they were offered randomization into the appropriate study by ER, MR, SW, TD, FM, GN, SA or AS. For those who agreed, the next numbered sealed envelope for their patient group was opened and the indicated treatment policy followed. The drug and dose of antiviral drug to be given to an individual patient was at the discretion of their attending physician (MH, J O’B, SM, GJ, or DT), but normally consisted of ganciclovir 5 mg per kilogram twice a day or valganciclovir 900 mg twice a day. As alternatives, foscarnet at 60 mg per kilogram three times a day, foscarnet at 90 mg per kilogram twice a day or half dose foscarnet plus half dose ganciclovir were permitted, having previously been shown to control CMV viraemia to a similar extent as full-dose ganciclovir.[5,7] All drug dosages given to individual patients were adjusted for renal function according to the manufacturers’ guidelines. This study thus assessed the policy of administering pre-emptive therapy without dictating which antiviral drug should be used. Clinical staff were not blinded to treatment assignment in this open label study, but the laboratory staff who produced the viral load measurements were. The two clinical trials are summarized below:

### Part A. To determine if immediate treatment of patients with two consecutive low levels of CMV viraemia significantly reduced the number who subsequently developed a viral load greater than 3000 genomes/ml blood

Patients with a single viral load measurement between 200 and 3,000 genomes/ml were monitored but were not treated. Those who developed a second, consecutive viral load between 200 and 3,000 genomes/ml were offered randomization to be treated immediately until two consecutive blood samples without detectable CMV DNA were obtained or to only start treatment if the viral load increased above 3000 genomes/ml. Because the availability of real-time PCR results was novel, there was no available data to guide the sample size required. Assuming that only 1% of those randomized to receive immediate treatment would develop a viral load greater than 3000 genomes/ml, a study size of 72 patients had 90% power to detect an increase to 25% among those randomized to monitor without treatment.

### Part B. To determine if stopping pre-emptive therapy before viraemia became undetectable increased the proportion of patients who developed a second episode of viraemia above 3000 genomes/ml

Any patient with a viral load measurement above 3,000 genomes/ml blood was treated as part of our standard clinical management protocol. Patients were then offered randomization to stop therapy when they had two consecutive viral load measurements below 200 genomes/ml or two consecutive viral load measurements below 3,000 genomes/ml. All patients were monitored to determine if the viral load subsequently increased above 3000 genomes/ml. The sample size calculation was based on the estimate that a second episode of replication requiring pre-emptive therapy (greater than 3000 genomes/ml) was expected in 30% of patients randomized to be treated until they had two consecutive viral load measurements below 200 genomes/ml. A study size of 106 patients had 90% power to detect a doubling of this incidence to 60%

### Objective and primary end-points

To define the number of patients with CMV viraemia who subsequently develop a viral load greater than 3000 genomes/ml in each part of the study.

The primary end-point of Part A compared statistically the proportion in each arm who developed viraemia greater than 3000 genomes/ml. The primary end-point of Part B compared statistically the proportion in each arm who subsequently developed viraemia greater than 3000 genomes/ml.

These objectives and primary end-points did not change during the study.

### Trial administration

The studies (protocol 6077) were approved by the Royal Free Hospital and Medical School Research Ethics Committee (institutional review board) on 20 November 2002. All patients gave informed consent for the study which was documented in writing. No children, or adults unable to give informed consent, were recruited into the study. The sponsor was University College London. The first patient was randomized in February 2003 and the last in December 2011. The study was registered in June 2009 as ClinicalTrials.gov NCT00947141 when registration of non-industry clinical trials became required, but patients had already been recruited by then. The authors confirm that all ongoing and related trials for cytomegalovirus have been registered before recruitment of the first patient. Patients who were excluded after randomization were replaced. Once a patient had been randomized into Part A or Part B, they could not subsequently be entered into the other part. Patients taking part in a randomized controlled trial of a prototype CMV vaccine at our institution[14] were not eligible for either Part A or Part B.

The regulations governing clinical trials in the UK changed in 2005. The Medicines and Healthcare Regulatory Authority provided an algorithm on their website to allow investigators to decide which ongoing studies would require a Clinical Trials Authorization under the new regulations. This study did not meet the criteria laid out in their algorithm and so no Clinical Trials Authorization was sought. During a site inspection in March 2007, inspectors from the Medicines and Healthcare Regulatory Authority agreed with that assessment using the algorithm, but asked for a Clinical Trials Authorization to be applied for nonetheless. Consequently, the study (both parts) was on hold from 20 April 2007 while the Medicines and Healthcare Regulatory Authority reviewed the Clinical Trials Authorization application and protocol. Of note, no changes were requested to the protocol, the Clinical Trials Authorization was provided and the study restarted on 26 October 2007.

### Statistical methods

Differences between viral loads were assessed by Student’s t-test. Differences between median durations of treatment were assessed by Mann-Whitney test. Time to events employed Kaplan-Meier analysis. Categorical analysis used Fisher’s exact test. All tests were two-sided and p-values ≤ 0.05 were regarded as significant. SAS version 9.3 (SAS Institute Inc, Cary, NC) was used.

## Results

**Fig 1:**
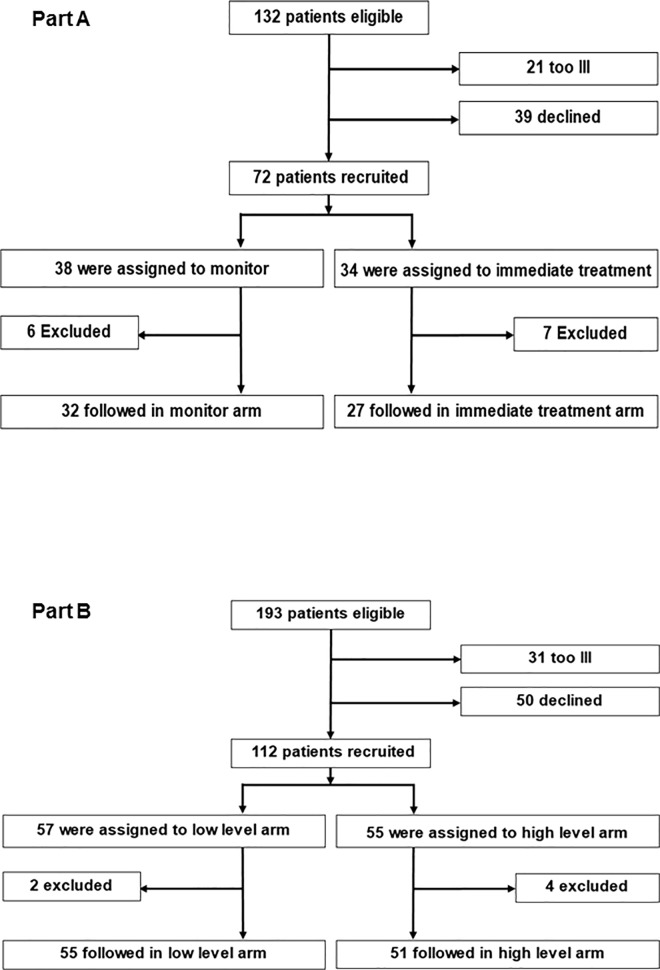
Disposition of patients in parts A and B of the study.

The disposition of all recruited patients is shown in Fig 1. The allocation of evaluable patients to different studies and arms and their demographic characteristics are shown in Table 1. No suspected unexpected serious adverse events were reported for any patient.

**Table 1:** Demographic characteristics of evaluable patients according to the study and randomized arm they were allocated to.

Characteristic	Part A		Part B	
	Monitor	Treat	2 <200	2 <3000
Number of patients	32	27	55	51
Transplant				
• Kidney	12	13	28	27
• Liver	12	8	16	14
• Bone marrow	8	6	11	10
High CMV risk				
• D+ R- SOT	3	2	16	17
• D- R+ BMT	2	2	3	3
Male	23	21	37	24
Female	9	6	18	27
Age				
• Median	49	52	50	51.5
• Range	19–76	17–74	17–67	22–71
Race				
• Caucasian	17	13	34	32
• Black	3	4	10	5
• Asian	11	8	7	8
• Hispanic	0	0	0	0
• Other	1	2	5	5

### Part A. Patients with a low level of CMV infection at risk of developing a viral load greater than 3000 genomes/ml

A total of 132 eligible patients were identified but 21 were considered by their clinicians to be too ill to discuss the study while 39 declined to enter. After randomization, 6 patients in the monitor arm and 7 in the immediate treatment arm were excluded. The reasons for this in the monitor arm were: two had a previous episode of CMV viraemia, one had been started on treatment already and three were lost to follow-up. The reasons for this in the immediate arm were: two had multiple transplants, one had a high level CMV viraemia, one patient request because of pill burden and three were lost to follow-up.

Part A stopped in December 2011 once recruitment to Part B was completed, because the sponsor was unwilling to continue to support the study given the slow rate of recruitment. At this time, 13 recruited patients had been excluded, so 59/72 (82%) of the target population had been recruited and followed. These 59 patients were randomized to monitor (n = 32) or to be treated immediately (n = 27) and constituted the modified intention-to-treat population for Part A. The median [range] post-transplant day of randomization was 44.5 [10–122] in the monitor arm and 44 [6–78] in the immediate treatment arm. The median post-transplant day of last sample collection was 102 [13–595] in the monitor arm and 93 [39–651] in the immediate treatment arm.

**Fig 2:**
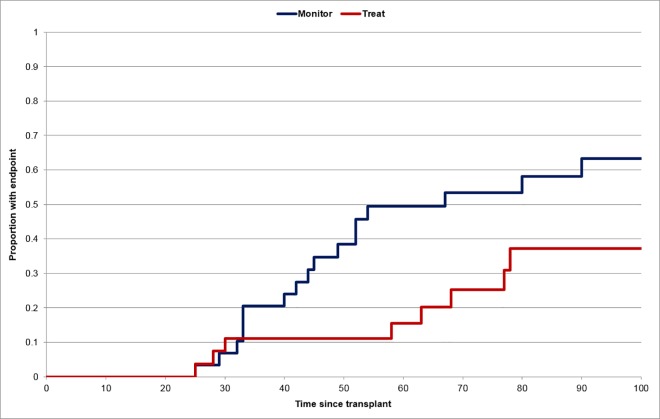
Days from transplantation to reaching an endpoint of viraemia greater than 3000 genomes/ml whole blood for patients in Part A of the study according to randomization to immediate treatment or to monitoring of viraemia.

The primary endpoint (a viral load of greater than 3000 genomes/ml) was observed in 28 patients (48%); 18/32 (56%) in the monitor arm versus 10/27 (37%) in the immediate treatment arm (p = 0.1925). The time from transplant to developing the primary endpoint was significantly longer (hazard ratio and 95% CI 2.52 (1.11, 5.73) p = 0.022) among those randomized to immediate treatment (Fig 2). Three patients were receiving treatment at the time their viral load increased above 3000 genomes/ml; the remaining patients in the immediate treatment arm had completed their therapy so were no longer receiving treatment when their viral load increased above 3000 genomes/ml. Of note, 3 patients in the immediate treatment arm and 3 in the monitor arm already had a viral load greater than 3000 genomes/ml in a blood taken at the time of randomization (although the result was not known at that time). Removal of these 6 cases as part of an “as treated” analysis produced results that remained statistically non-significant (15/29(52%) versus 7/24(29%); p = 0.1610). All the treated patients in Part A received ganciclovir or valganciclovir with none receiving foscarnet.

Although the study was not powered to provide results for each patient group separately, we performed exploratory analyses to determine if there were marked differences for patients in the high risk groups (Table 1) or for bone marrow transplant patients. The primary endpoint occurred in 6/28 (21%) high-risk patients compared to their prevalence in Part A (Table 1) of 9/59 (15%) patients. Bone marrow transplant patients experienced 9/28 (32%) endpoints compared to their prevalence in Part A (Table 1) of 14/59 (24%) patients.

Only one patient in Part A developed CMV end-organ disease. A bone marrow transplant patient became PCR positive on post-transplant days 71 and 77 (both less than 3000 genomes/ml) and so was recruited into the monitor arm of Part A. On day 102, symptoms of vomiting prompted endoscopy. Gastric and duodenal biopsies had high CMV viral loads by PCR, 135,618 and 35,476 genomes/microgram of DNA respectively, but no inclusion bodies were seen by histopathology or by immunostaining so a diagnosis of presumptive gastrointestinal disease was made. None of six blood samples taken between recruitment and biopsy had a viral load greater than 3000 genomes/ml. His viral load became undetectable after a three week course of intravenous ganciclovir after which repeat gastric and duodenal biopsies contained no CMV DNA detectable by PCR. He was then given prophylaxis with valaciclovir to avoid the bone marrow toxicity of valganciclovir. By day 200, his viral load became detectable above 3000 genomes/ml and valganciclovir, limited by bone marrow toxicity, failed to bring this to undetectable levels. After another 100 days of continuing viraemia he developed retinitis which was confirmed by PCR from a vitreous sample. Thus, this patient had proven CMV end organ disease almost one year post-transplant and approximately 7.5 months after randomization into the study.

### Part B. Patients at risk of developing a second episode of a viral load above 3000 genomes/ml after therapy had been discontinued at one of two defined viral load cut-offs

A total of 193 eligible patients were identified but 31 were considered by their clinicians to be too ill to discuss the study while 50 declined to take part (Fig 1). 112/112 (100%) of the target number of patients were recruited, including replacements for 6 who were excluded (see below). Of these, 57 were randomized to stop treatment once two levels below 200 genomes/ml had been detected and 55 patients were randomized to stop treatment once two levels below 3000 genomes/ml had been detected. After randomization, two patients in the low-level arm and four in the high-level arm were excluded. The reasons for this in the low-level arm were: one had multiple transplants, one patient request to avoid venepuncture. The reasons for this in the high-level arm were: two had a previous episode of CMV viraemia, one had multiple transplants and one did not receive the allocated drug. Thus, 55 and 51 patients formed the modified intention-to-treat population for Part B. The median [range] post-transplant day of randomization was 41 [5–192] in the less than 200 arm and 38 [13–165] in the less than 3000 arm. The median post-transplant day of last sample collection was 128 [33–649] in the less than 200 arm and 162 [41–737] in the less than 3000 arm.

The patients were well matched, because there was no significant difference (p = 0.8) in the peak viral load of the initial episode among patients randomized to two levels less than 200 genomes/ml (mean peak = 4.01± 0.52 log10 genomes/ml) compared to those randomized to two levels less than 3000 genomes/ml (mean peak = 4.04 ± 0.57 log10 genomes/ml). There was also no significant difference in the doubling time (mean 1.96 ± 1.15 days vs 1.64 ± 1.03 days; p = 0.12) or the half-life of decline (mean 2.08 ± 1.08 days vs 2.03 ± 1.14 days; p = 0.89) between the two groups.

The primary endpoint of a subsequent episode of viral replication greater than 3000 genomes/ml blood was reached in 42 patients; 19/55 (35%) randomized to two levels less than 200 genomes/ml and 23/51 (45%) randomized to two levels less than 3000 genomes/ml (Table 2); this difference was not statistically significant (p = 0.322); estimate of difference = 11% (95% CI -8%, 29%). As expected, the duration of pre-emptive therapy was significantly shorter in patients randomized to stop treatment on the basis of two consecutive PCR values below 3000 genomes/ml compared to two viral loads less than 200 genomes/ml (median days of treatment 30 [range 8–121] versus 24 [7–62] p = 0.0012).

**Table 2:** Number of patients in Part B of study with episodes of high or low level viraemia.

Randomized Arm	Number of patients with episodes of viraemia				Totals
	High and Low	High	Low	Neither	
2 x <200	9	9	18	19	55
2 x <3000	11	12	11	17	51
**Totals**	20	21	29	36	106

**Fig 3:**
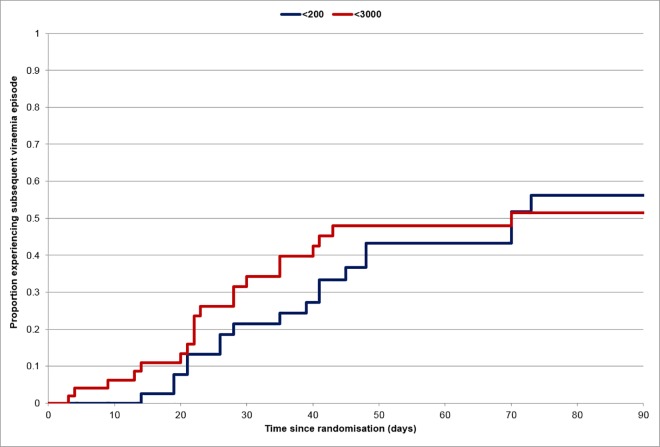
Proportion of patients experiencing a subsequent viraemic event requiring treatment according to whether initial therapy was stopped after two levels below 3000 genomes/ml or two levels below 200 genomes/ml.

When the number of days of treatment for subsequent episodes were included, the overall duration of pre-emptive therapy given to the two sets of patients was no longer significant (median days of treatment 38 [8–347] versus 32 [7–392]; p = 0.33). Because each median value had increased by 8, we suspected this was because of the expanded range of values seen, but explored the results to ensure that patients stopping therapy earlier had not been predisposed to have additional episodes of viraemia. The median days of treatment amongst those who needed any subsequent treatment was 27 [range 12–308] versus 33 [10–336]; p = 0.22). Several patients developed more than one subsequent episode of viraemia, some with viral loads greater than 3000 genomes/ml. The number of patients who had subsequent episodes of high level viraemia, low level viraemia, both or neither are shown in Table 2, while the number of such episodes is shown in Table 3. Overall, there was no difference between patients according to their randomized arm. The recurrences of patients randomized to stop pre-emptive therapy early appeared earlier, but the difference was not statistically significant (hazard ratio and 95% CI 1.22 (0.65, 2.26) p = 0.53; Fig 3).

**Table 3:** Number of episodes of viraemia experienced by patients in Part B of the study.

Randomized Arm	Number of discrete episodes of viraemia per patient							
	High level >3000				Low level <3000			
	1	2	3	>3	1	2	3	>3
2 x <200 genomes/ml	16	0	1	1	18	5	1	3
2 x <3000 genomes/ml	17	2	2	2	14	5	3	0

We explored potential clinico-pathological links between viral load measurements and outcomes. The median nadir viral load was 1.25 log10 genomes/ml for those randomized to two levels less than 200 genomes/ml, which was significantly (p = 0.03) lower than that (1.60 log10 genomes/ml) in those randomized to two levels less than 3000 genomes/ml. Among those with second episodes above 3000 genomes/ml, there was no significant difference (p = 0.9) in the viral load of the initial peak of those randomized to two levels less than 200 genomes/ml (4.01 log10 genomes/ml) compared to those randomized to two levels less than 3000 genomes/ml (4.06 log10 genomes/ml). There was also no significant difference in doubling time (1.88 vs 1.75 days; p = 0.6) or half-life of decline (1.94 vs 2.12 days; p = 0.18) in the two groups. However, within each group, the median viral load of the initial peak was significantly greater than that of the subsequent peak (4.61 vs 3.99; p<0.001 for those randomized to two levels less than 200 genomes/ml and 4.74 vs 3.84 log10 genomes/ml; p = 0.015 for those randomized to two levels less than 3000 genomes/ml). All the treated patients received ganciclovir or valganciclovir except for six (2 in the less than 3000 arm and 4 in the less than 200 arm) who received foscarnet. There was no obvious difference in the viral load parameters of those who received foscarnet.

Although the study was not powered to provide results for each patient group separately, we performed exploratory analyses to determine if there were marked differences for patients in the high risk groups (Table 1) or for bone marrow transplant patients. The primary endpoint occurred in 18/42 (43%) high-risk patients compared to their prevalence in Part B (Table 1) of 39/106 (37%) patients. Bone marrow transplant patients experienced 5/42 (12%) endpoints compared to their prevalence in Part B (Table 1) of 21/106 (20%) patients.

No patients in Part B developed CMV end organ disease.

## Discussion

This manuscript describes the first assessment of a policy of when to start and when to stop pre-emptive therapy using a randomized clinical trial design. For Part A of the study, it is clear from Fig 2 that immediate treatment of some patients decreased their risk of having a viral load greater than 3000 genomes/ml. However, in many cases this acted to delay, rather than to eliminate, the patients' difficulty in controlling CMV replication. It might be argued that treatment had not been given a fair chance to work in 3 patients in the immediate treatment arm of Part A, because they already had a viral load greater than 3000 genomes/ml when they were randomized. However, our study addressed the policy of whether or not treatment should be given to such patients and the results illustrate that very little time is available to institute effective therapy in patients with two low level PCR results if the objective is to stop the viral load rising above 3000 genomes/ml. In addition, 3 patients had an increase in viral load while they were still taking ganciclovir, showing that administration of therapy does not always immediately control CMV replication in immunocompromized hosts–a phenomenon that has been previously described by us and others.[11,15–16] To put this in perspective, without receiving antiviral therapy, the immune system of 14/32(44%) patients randomized to the monitor arm managed to prevent their viral load increasing above 3000 genomes/ml, a figure which was increased, non-significantly, to 17/27(63%) for those randomized to immediate treatment. Based on these results, we cannot recommend that the current criteria for starting pre-emptive therapy are changed for patients with two consecutive low level PCR results.

In Part B of the study, patients randomized to stop treatment before viraemia became undetectable (below 200 genomes/ml blood) needed a significantly shorter duration of pre-emptive therapy, so the objective of reducing exposure to antiviral drugs was achieved. However, this potentially beneficial effect was attenuated on follow-up. Based on these results, we cannot recommend that the current criteria for stopping pre-emptive therapy are changed. Indeed, the significantly higher nadir viral load in those given pre-emptive therapy until they had two blood samples with CMV DNA below 3000 genomes/ml suggests that the initial infection may have been inadequately treated to represent a focus from which viraemia could recur. Thus, despite the significantly shorter initial episode of treatment, we cannot recommend that treatment is stopped, as a matter of routine, at the earlier time point chosen here. However, if an individual patient experiences side-effects from pre-emptive therapy, then our results suggest that stopping this once the viral load falls below 3000 genomes/ml on two occasions is likely to have only minimal effects on the overall outcome.

All of the other measurements of viral load described here are consistent with those we have reported previously, emphasizing that these represent robust viral biomarkers of the natural history of CMV infection post transplantation in both solid organ and stem cell transplant recipients.[4,7,8,11,12] The viral load parameters of the initial episode could not distinguish those who were destined to have a subsequent episode of viraemia so there seems to be little prospect of using these parameters to predict who is at future risk. However, confirmation of the earlier observation that the peak viral load of a subsequent episode is significantly lower than that of the initial episode is consistent with CMV infection acting as an endogenous immunogen to stimulate the immune system.[17] This hypothesis could be tested by measuring such responses directly[18] and has the potential to identify correlates of protective immunity able to inform the selection of antigenic components for future prototype vaccines.

The strengths of this single centre study include the use of validated assays of CMV viral load and detailed biomathematical analyses. Weaknesses include the relatively small sample size and the long time required to recruit patients. Nevertheless, in the absence of any licensed new antiviral drug with activity against CMV, we believe the results are relevant to contemporary medical practice. We also show that, despite differences in CMV natural history,[8,19] patients from three different transplant groups can be recruited into a single protocol provided that they meet virologically-defined entry criteria and are stratified by organ transplanted. Overall, the results from the two randomized controlled trials described here underscore the vulnerable nature of transplant patients to CMV infection and their ongoing risks for repeat infection after an initial episode has been treated. They emphasize that improved ways of helping the immune system to control CMV infection are required in the way that some patients cannot control CMV replication once prophylaxis with valganciclovir has been stopped, even if given for 200 days post transplant.[3] Thus, we reported on a separate cohort of patients who had reduced viraemia and need for valganciclovir treatment post-transplant after pre-transplant administration of an investigational CMV vaccine compared to controls randomized to receive placebo.[14] Vaccines able to induce immunity in seronegative individuals and to boost the natural immunity of seropositives may therefore represent a better way of controlling CMV replication than comparing different policies of pre-emptive therapy in those patients who fail to keep CMV suppressed to undetectable levels post-transplant.[14,20] Indeed, the natural history data presented here suggest the additional possibility that administration of vaccine or placebo at the time of detecting a single, low level PCR positive result post transplant represents an alternative study design to identify vaccines able to enhance immunological control of CMV in these patients.

## Supporting Information

S1 File Protocol version 5.0. (PDF)

S2 File CONSORT checklist. (PDF)
