# Correction: Randomized Controlled Trials to Define Viral Load Thresholds for Cytomegalovirus Pre-Emptive Therapy

**DOI:** 10.1371/journal.pone.0185298

**Published:** 2017-09-19

**Authors:** Paul D. Griffiths, Emily Rothwell, Mohammed Raza, Stephanie Wilmore, Tomas Doyle, Mark Harber, James O’Beirne, Stephen Mackinnon, Gareth Jones, Douglas Thorburn, Frank Mattes, Gaia Nebbia, Sowsan Atabani, Colette Smith, Anna Stanton, Vincent C. Emery

An affiliation for the last author is missing. Vincent C. Emery is also affiliated with Department of Microbial and Cellular Sciences, University of Surrey, Guildford, Surrey, United Kingdom.
