# Supplementary material for: Randomized Controlled Trials to Define Viral Load Thresholds for Cytomegalovirus Pre-Emptive Therapy
Source: PLoS One. 2016 Sep 29;11(9):e0163722. doi: 10.1371/journal.pone.0163722 (PMC5042415; doi:10.1371/journal.pone.0163722)
Supplement: S1 File — (PDF) [file pone.0163722.s001.pdf]

Determining a Viral Load Threshold for Pre-emptive Therapy for Cytomegalovirus Infection in Transplant Patients Using Real Time PCR Monitoring

Short Title:
Determining a viral load threshold for treating CMV

Chief Investigator:

Professor Paul Griffiths
Department of Virology
Royal Free & University College Medical School
Pond Street
London NW3 2QG
Tel: 0207 830 2997
Fax: 0207 830 2854
Email: p.griffiths@medsch.ucl.ac.uk

Principal Investigators:

Professor Steve Mackinnon
Dept of Haematology
Royal Free & University College Medical School
Pond Street
London NW3 2QG

Dr P. Sweny
Dept of Nephrology
Royal Free & University College Medical School
Pond Street
London NW3 2QG

Professor A. K. Burroughs
Hepatobiliary Medicine and Liver Transplant Unit
Royal Free & University College Medical School
Pond Street
London NW3 2QG

Study Co-ordinator
Dr Sowsan Atabani
Department of Virology
Royal Free Hospital
Pond Street
London NW3 2QG

Tel: 0207 472 6403
Fax: 0207 830 2854
Email: s.atabani@medsch.ucl.ac.uk

Sponsor:
Royal Free R&D (code 6077)
Pond Street
London
NW3 2QG

Version number 4.0
31 May 2007

SIGNATURE PAGE

The signatures below constitute the approval of this protocol and the attachments, and provide the necessary assurances that this trial will be conducted according to all stipulations of the protocol, including all statements regarding confidentiality, and according to local legal and regulatory requirements and ICH guidelines.

Signed: ______________________________ Date: ______________

Chief Investigator: Paul Griffiths, MD

Signed: ______________________________ Date: ______________

Principal Investigator: Stephen Mackinnon MD

Signed: ______________________________ Date: ______________

Principal Investigator: Paul Sweny, MD

Signed: ______________________________ Date: ______________

Principal Investigator: Andrew K Burroughs, MD

Table of Contents

1. Protocol Summary………………………………………………………. Page: 5

2. Background and Study Rationale…………………………………... Page: 6

3. Objectives……………………………………………………………… Page: 6
   3.1 Primary Objectives
   3.2 Secondary Objectives

4. Study Design………………………………………………………….. Page: 6

5. Patient Selection……………………………………………………… Page: 7
   5.1 Inclusion Criteria
   5.2 Exclusion Criteria

6. Study Procedure and Assessments………………………………… Page: 7
   6.1 CMV Monitoring
   6.2 Biochemistry and Haematological Monitoring
   6.3 Immune Function
   6.4 Treatment

7. Assessment of Safety………………………………………………… Page: 8

8. Ethical and Regulatory Aspects…………………………………….. Page: 9

9. Statistical Considerations……………………………………………. Page: 9

10. References…………………………………………………………….Page: 10

1. Protocol Summary

Participating Centre
Royal Free Hospital
Pond St
London NW3 2QG

Indication
All stem cell, renal, and liver transplant recipients with CMV viraemia

Study Design
Open Label Randomised Trial

Sample Size
Group A: (Low level CMV infection)
72 Patients

Group B (Patients receiving pre-emptive therapy)
106 Patients

Primary end point
The number of patients in whom viral load increases above 3,000 copies/ml (requires a second discrete episode from patients in group B).

2. Background and Study Rationale

In transplant recipients with CMV infection, the risk of developing CMV disease is directly proportional to the CMV DNA viral load. Historically at this Institution, patients were given preemptive therapy on the basis of two consecutive positive CMV PCR results as detected by a qualitative PCR technique. With the introduction of real time PCR, using a Taqman probe and the ABI7700 thermal cycler, it is possible to obtain rapid and sensitive results of viral load on clinical samples with a lower limit of detection of 200 copies/ml. Thus, viral load data can be incorporated into the clinical management of the patient.
From our natural history data, it has been shown that patients with CMV disease had a CMV PCR load ranging from 14,000 to 203 million (median 175,500). The lower bound of the 95% confidence limits of this distribution was 37,000 copies/ml and we aimed to initiate therapy in time to prevent CMV viral load reaching this value. To give a margin of safety, bearing in mind the 1 day average doubling-time of CMV and the timing of sampling twice-weekly, we therefore recommended that preemptive therapy be given once the viral load increases above 3,000 copies/ml. In the past, all patients with a CMV PCR load between 200 and 3,000 copies/ml have received preemptive treatment because the previous PCR assay did not give a quantitative result. As treatment is associated with side effects such as neutropaenia (ganciclovir) and renal impairment (foscarnet) it would be preferable to avoid unnecessary exposure where possible. This study aims to determine: a) whether those patients with 'low level' viral load results (between 200 and 3,000 copies/ml) could be monitored as opposed to starting preemptive therapy with valganciclovir, ganciclovir and/or foscarnet; b) whether those patients with 'high level' viral load results (above 3,000 copies/ml) could stop preemptive therapy earlier, thus maximising the benefits of therapy and minimising its risks.

3. Objectives

3.1 Primary Objectives

- 3.1.1 To define the number of patients in Group A with a low level of CMV reactivation who subsequently develop a viral load greater than 3000 copies/ml
- 3.1.2 To define the number of patients in Group B who develop a second episode of a viral load above 3000 copies/ml after therapy has been discontinued at the defined viral load cut-offs.

3.2 Secondary Objectives

- 3.2.1 To define the duration of antiviral therapy needed to treat CMV viraemia
- 3.2.2 To record the rate of increase in viral load prior to starting preemptive therapy
- 3.2.3 To correlate viral loads with CMV specific immune function

4. Study Design

An open label randomised trial.
Monitoring for CMV viraemia will be performed by PCR twice weekly as part of routine clinical care.
In patients who agree to enter the study, blood samples will be taken for immunological monitoring on two occasions before becoming PCR negative.
Randomisation will be by randomisation tables. Envelopes containing randomisation codes will be kept in the virology department, and supplied to the research nurse/ transplant clinician at the time of enrolment.

Group A:
Patients with two consecutive CMV viral loads between 200 and 3000 copies/ml in the renal, liver and stem cell groups will be randomised to receive immediate pre-emptive therapy or continue with frequent CMV PCR monitoring twice weekly, commencing pre-emptive therapy only if viral load exceeds 3000 copies/ml.

Group B:
Patients with CMV infection with viral loads greater then 3000 copies/ml who are receiving pre-emptive therapy will be randomised to have therapy discontinued when their viral load falls below 3000 copies/ml on two consecutive occasions or therapy discontinued when they have two consecutive CMV negative PCR results (ie below 200 copies/ml).

5. Patient Selection

5.1 Inclusion Criteria

- 5.1.1 All Stem Cell, Renal and Liver Transplant recipients
- 5.1.2 Wiling to give informed consent
- 5.1.3 A) All patients with CMV viraemia (between 200 and 3000 copies/ml) in the liver, renal and stem cell groups in two consecutive samples.
  B) Those patients requiring pre-emptive therapy because viral load is > 3,000 copies/ml
- 5.1.4 All patients in either section of the study must be available for CMV PCR monitoring at least twice per week

5.2 Exclusion Criteria

- 5.2.1 Profound neutropaenia considered to preclude administration of ganciclovir or profound renal failure considered to preclude administration of foscarnet
- 5.2.2 Inability to give informed consent
- 5.2.3 In the stem cell group, Donor negative, Recipient negative transplants.
- 5.2.4 In the stem cell group: matched unrelated donors who are CMV seronegative
- 5.2.5 Those patients who have been in Group A cannot then enter the Group B part of the study.
- 5.2.6 Those patients who have been in Group B cannot then enter the Group A part of the study.

6. Study Procedure and Assessments

6.1 CMV Monitoring

All patients will be monitored by citrated or EDTA peripheral blood samples for CMV PCR viral load twice weekly as part of routine clinical care. Personnel performing the CMV PCR viral load monitoring will be blinded to which arm of the study the patient is on.

6.2 Biochemistry and Haematological Monitoring

All patients recruited to the trial will have routine full blood counts, biochemistry and renal function as part of clinical care and the results will be used by the clinicians to adjust the doses of preemptive therapy.

6.3 Immune Function

24 ml Blood will be taken on two occasions before becoming PCR negative to monitor for CMV-specific immune function, including ELISPOT, intracellular cytokine assay and CMV specific MHC Class I tetramers.

6.4 Treatment

Patients will be prescribed preemptive therapy at the discretion of the responsible clinician, using drugs and dosages appropriate for each patient. For example, full doses include ONE of the following: valganciclovir 900mg bd; OR ganciclovir 5mg/Kg iv bd; OR foscarnet 90mg/Kg iv bd; OR foscarnet 60mg/Kg tds; OR ganciclovir 5mg/Kg iv od PLUS foscarnet 90mg/Kg iv od. In all cases, doses will be reduced according to estimated creatinine clearance (foscarnet, valganciclovir and ganciclovir) or neutropenia (valganciclovir and ganciclovir).

7. Assessment of Safety

Adverse events and serious adverse events occur frequently in these seriously ill patients, unrelated to the use of ganciclovir or foscarnet and these will not be recorded or reported. Transplant patients are discussed at weekly, multi-disciplinarily ward rounds and their medical history will be reviewed for any occurrences of serious adverse events or SUSARs (see definitions below) which are deemed attributable to the use of preemptive therapy. Any such SAEs or SUSARs will be reported to the sponsor, the ethics committee and the MHRA using a standard form.

Definition of an Adverse Event

An AE is any untoward medical occurrence in a subject to whom a medicinal product has been administered including occurrences which are not necessarily caused by or related to that product.

An AE can therefore be any unfavourable and unintended sign, symptom or disease temporally associated with the use of a medicinal product, whether or not considered related to the medicinal product.

The worsening of an existing sign of symptom is also considered an AE.

Definition of a Serious Adverse Event

Serious and severe are not synonymous. The term severe is often used to describe the intensity (severity) of a specific event (as in mild, moderate, or severe myocardial infarction); the event itself, however, may be of relatively minor medical significance (such as severe headache). This is not the same as serious which is based on patient/event outcome or action criteria usually associated with events that pose a threat to a patient's life or functioning. Seriousness, not severity, serves as a guide for defining regulatory reporting obligations.

An SAE is any untoward medical occurrence that:

- Results in death
- Is life-threatening
- Requires inpatient hospitalization or prolongation of existing hospitalization
- Results in persistent or significant disability/incapacity
- Is a congenital anomaly/birth defect
- Is an important medical event

Suspected Serious Adverse Reaction (SSAR):

This is an adverse reaction that is classed in nature as serious and which is consistent with the information about the medicinal product set out in the Summary of Product Characteristics.

Suspected Unexpected Serious Adverse Reaction (SUSAR)
This is an adverse reaction that is classed in nature as serious and which is not consistent with the information about the medicinal product set out in the Summary of Product Characteristics.

8. Ethical and Regulatory Aspects

Conduct of Study
The study will be conducted according to ICH Harmonised Tripartite Guideline for Good Clinical Practice as came into effect in Europe on 17th January 1997. The principles of the Declaration of Helsinki will also be observed.

Informed Consent
It is the investigator's responsibility to obtain the written informed consent from the patient. Participants must have read and understood the information sheet. Participants will be given a copy of the signed information sheet and consent form.

Ethics committee
The study will be submitted to the Local Research Ethics Committee for approval. The study may not commence until fully documented approval has been received. The investigator will notify the LREC of any amendments to the protocol.

9. Statistical Considerations

The primary end point is the number of patients in whom viral load increases above 3,000 copies/ml (requires a second discrete episode from patients in group B).

9.1 Sample size considerations.

For Group A, our historical data show that no patients developed CMV disease when they were treated with low viral loads. If we assume that only 1% will develop viral loads >3000 copies/ml after starting treatment and that a rate of 25% or more requiring treatment among those allocated to monitoring would be a failure, then a study size of 72 (36 + 36) patients has 90% power to detect this difference ($\alpha<0.05$).

For Group B, our historical data show that 30% of patients require a second course of preemptive therapy. This trial would be a failure if this proportion doubled to 60% or more. A study size of 106 (53 + 53) patients has 90% power to detect this difference ($\alpha<0.05$).

The data will therefore be analysed when 72 patients have been recruited and followed in Group A and 106 recruited and followed in Group B. No interim analyses are planned.

10. References

1. Emery VC, Sabin CA, Cope AV, Gor D, Hassan-Walker AF, Griffiths PD. Application of viral load kinetics to identify patients who develop cytomegalovirus disease after transplantation. *Lancet* (2000),355,2032-2036.

2. Mattes FM, Hainsworth EG, Geretti AM, Nebbia G, Prentice G, Potter M, Burroughs AK, Sweny P, Hassan-Walker AF, Okwuadi S, Sabin C, Amooty G, Brown VS, Grace SC, Emery VC, Griffiths PD. A randomized, controlled trial comparing ganciclovir to ganciclovir plus foscarnet (each at half dose) for preemptive therapy of cytomegalovirus infection in transplant recipients. *Journal of Infectious Diseases*. (2004), 189, 1355-61.

3. Mattes FM, Hainsworth EG, Hassan-Walker AF, Burroughs AK, Sweny P, Griffiths PD, Emery VC. Kinetics of cytomegalovirus load decrease in solid-organ transplant recipients after preemptive therapy with valganciclovir. *Journal of Infectious Diseases*. (2005), 191, 89-92.
